# Synchronous Periampullary Tumors in a Patient With Pancreas Divisum and Neurofibromatosis Type 1

**DOI:** 10.3389/fgene.2020.00395

**Published:** 2020-04-28

**Authors:** Cleandra Gregório, Clévia Rosset, Laura da Silva Alves, Cristina Brinkmann Oliveira Netto, Simone Marcia dos Santos Machado, Vivian Pierri Bersch, Alessandro Bersch Osvaldt, Patricia Ashton-Prolla

**Affiliations:** ^1^Laboratório de Medicina Genômica, Centro de Pesquisa Experimental, Hospital de Clínicas de Porto Alegre, Porto Alegre, Brazil; ^2^Programa de Pós-graduação em Genética e Biologia Molecular, Universidade Federal do Rio Grande do Sul, Porto Alegre, Brazil; ^3^Faculdade de Medicina, Universidade Federal do Rio Grande do Sul, Porto Alegre, Brazil; ^4^Serviço de Genética Médica, Hospital de Clínicas de Porto Alegre, Porto Alegre, Brazil; ^5^Serviço de Patologia, Hospital de Clínicas de Porto Alegre, Porto Alegre, Brazil; ^6^Serviço de Cirurgia do Aparelho Digestivo, Grupo de Vias Biliares e Pâncreas, Hospital de Clínicas de Porto Alegre, Porto Alegre, Brazil; ^7^Grupo do Pâncreas, Serviço de Cirurgia do Aparelho Digestivo, Hospital Moinhos de Vento, Porto Alegre, Brazil; ^8^Programa de Pós-graduação em Medicina: Ciências Cirúrgicas, Universidade Federal do Rio Grande do Sul, Porto Alegre, Brazil; ^9^Faculdade de Medicina, Universidade Federal do Rio Grande do Sul, Porto Alegre, Brazil

**Keywords:** synchronous neoplasms, GIST, periampullary tumors, neurofibromatosis type 1, pancreas divisum, *CFTR* pathogenic variant, *NF1* pathogenic variant

## Abstract

**Introduction:**

In this study, we describe for the first time a Neurofibromatosis type 1 patient with pancreas divisum, multiple periampullary tumors and germline pathogenic variants in *NF1* and *CFTR* genes.

**Case report:**

A 62-year-old female NF1 patient presented with weakness, choluria, nausea, and diffuse abdominal pain to an emergency room service. Magnetic resonance imaging revealed an abdominal mass involving the periampullary region and pancreas divisum. After surgical resection, three synchronous neoplasms were detected including two ampullary tumors (adenocarcinoma of the major ampulla and a neuroendocrine tumor of the minor ampulla) and a gastrointestinal stromal tumor (GIST). Germline multigene panel testing (MGPT) identified two pathogenic heterozygous germline variants: *NF1* c.838del and *CFTR* c.1210-34TG[12]T[5].

**Conclusion:**

This is the first report of a Neurofibromatosis type 1 patient with pancreas divisum and multiple periampullary tumors harboring pathogenic germline variants in *NF1* and *CFTR* genes. The identification of two germline variants and a developmental anomaly in this patient may explain the unusual and more severe findings and underscores the importance of comprehensive molecular analyses in patients with complex phenotypes.

## Introduction

Pancreas divisum is a pancreatic duct developmental anomaly with an incidence of 4.5% (Dimitriou et al., 2018). The anomaly is caused by absent or incomplete fusion of the ventral (main or Wirsung) and dorsal (marginal or Santorini) ducts (Kanth et al., 2014) and results in coexistence of two ampullary systems: the ventral duct drains the pancreatic head through the major ampulla, while the dorsal duct drains the pancreatic body and tail through the minor ampulla (Ferri et al., 2019).

Ampullary neoplasms are rare, comprising 7% of all periampullary malignancies (Adsay et al., 2012), and association of theses tumors with pancreas divisum is considered an episodic event (Singh et al., 2003; Outtas et al., 2004; Kim et al., 2010). Their occurrence has been reported in families with hereditary cancer syndromes, such as Familial Adenomatous Polyposis (Pérez-Cuadrado-Robles et al., 2019) and Neurofibromatosis type 1 (NF1) (Tewari et al., 2014).

Neurofibromatosis type 1 (OMIM 162200) is one of the most common autosomal dominant disorders (incidence estimated at 1 in 2,500–3,000 live births) (Ferner et al., 2007). It is characterized by an increased risk of developing benign and malignant tumors, and a cumulative cancer risk of 20% in affected patients older than 50 years (Friedman and Birch, 1997; Upadhyaya, 2011). The *NF1* gene product, neurofibromin, functions as a GTPase activating protein for RAS but has no Ras-GTPase activity, which regulates cell proliferation and differentiation (Cawthon et al., 1990; Boyd et al., 2009; Tewari et al., 2014). Germline heterozygous loss-of-function mutations in the *NF1* gene lead to protein dysfunction and consequently, uncontrolled cell proliferation which has been associated with several of the NF1 clinical features. Loss of heterozygosity (LOH) has been reported as a necessary step for the development of malignancies in NF1 patients (Ruggieri and Packer, 2001). About one quarter of NF1 patients also have gastrointestinal involvement (Agaimy et al., 2012), with the occurrence of gastrointestinal stromal tumors (GISTs) and an increased incidence of neuroendocrine tumors (NET). The most frequent are somatostatin secreting duodenal NETs usually located in the periampullary region (Klein et al., 1989; Nunobe et al., 2003; Relles et al., 2010), followed by pheochromocytomas (Kalff et al., 1982) and pancreatic endocrine tumors (Thannberger et al., 2001; Fujisawa et al., 2002; Perren et al., 2006). A few NF1 patients with co-occurrence of GIST and NETs have been described in the literature (Tewari et al., 2014; Thavaraputta et al., 2019).

In this study, we describe and perform genetic analysis in a neurofibromatosis type 1 patient with pancreas divisum and multiple periampullary tumors. Although pancreas divisum is not a feature of NF1, the synchronous occurrence of multiple tumors in a NF1 background is extremely rare but more frequent than observed in the general population.

## Case Presentation

A 62-year-old Caucasian female patient with NF1 presented to the emergency room with symptoms of weakness, nausea, vomiting, choluria, and diffuse abdominal pain. She reported a previous diagnosis of pheochromocytoma and breast cancer. Upon physical examination, the patient was jaundiced and had a palpable and painful gallbladder, and multiple neurofibromas in the abdomen and extremities. Initial laboratory findings showed unusually high levels of bilirubin (total bilirubin 9.9 mg/dl and direct bilirubin 8.4 mg/dl), elevated serum C-reactive protein levels (29.8 mg/dl) and elevated liver enzymes.

**FIGURE 1:**
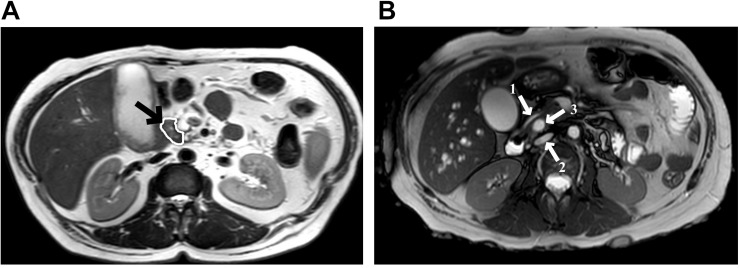
Abdominal magnetic resonance imaging. **(A)** T2 sequences show a small nodular mass (arrow) with a hypointense signal at the level of the major duodenal ampulla, measuring approximately 10.2 mm in its largest axis; **(B)** Pancreas divisum diagnosis, arrow 1 shows the ventral pancreatic duct, arrow 2 shows the dorsal pancreatic duct, and between those, the arrow 3 shows the common bile duct.

Due to the high levels of bilirubin, the patient was submitted to an endoscopic retrograde cholangiopancreatography for placement of a biliary stent and during the procedure, an ulcerated expansive periampullary lesion was identified and biopsied. Pathology examination of the biopsy specimen revealed a moderately differentiated adenocarcinoma. During further investigation, an abdominal magnetic resonance imaging showed not only a small hypointense nodular mass (T2 sequence) in the periampullary region of the duodenum, but also pancreas divisum (Figures 1A,B).

**FIGURE 2:**
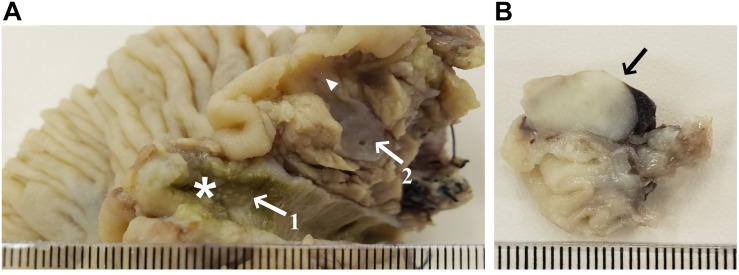
Macroscopy specimens of pylorus-preserving pancreaticoduodenectomy. **(A)** Pancreas divisum, arrow 1 shows the dorsal duct with a vegetative lesion (17.0 × 2.0 mm) extending to the major ampulla (star); arrow 2 shows the ventral duct and the arrowhead shows a poorly defined densification area near the minor ampulla (5.0 mm); **(B)** Duodenal wall mass (12.0 mm).

**FIGURE 3:**
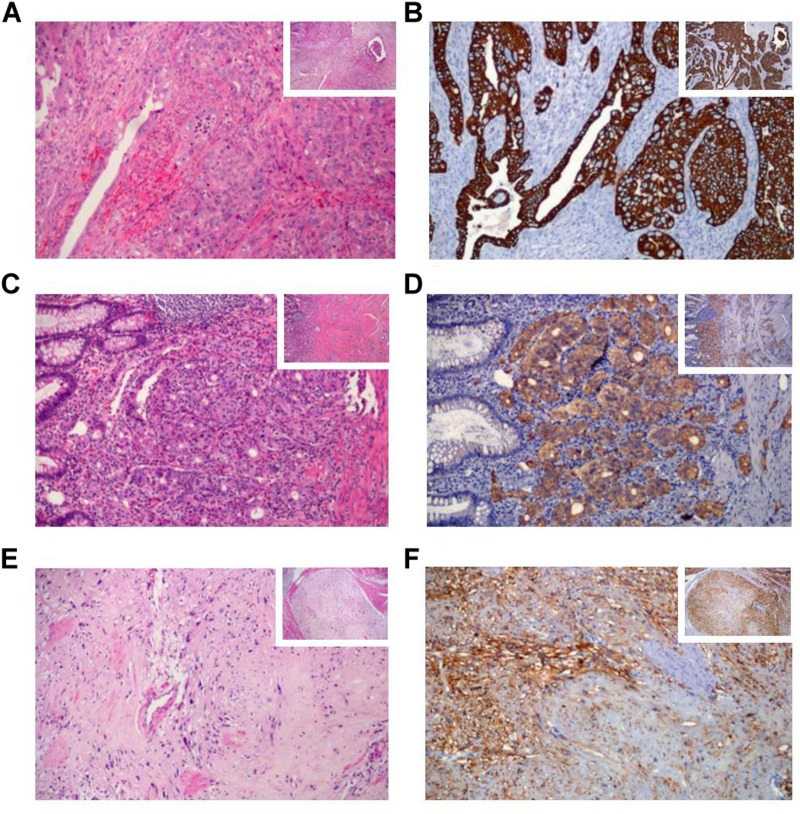
Histological and immunohistochemical features of the synchronic gastrointestinal tumors by hematoxylin-eosin (H&E) and immunohistochemical (IHC) staining. **(A)** The poorly-differentiated adenocarcinoma of the major ampulla H&E **(B)** and CK7 IHC staining. **(C)** The well-differentiated neuroendocrine tumor of the minor ampulla H&E, **(D)** and synaptophysin IHC staining. **(E)** Spindle cells in the gastrointestinal stromal tumor H&E, **(F)** and DOG1 IHC staining. The histological sections stained are presented at x100 magnification and the right small squares represent ×40 magnification.

The patient underwent a resection of the proximal jejunum, pylorus-preserving pancreaticoduodenectomy (PPPD) with routine reconstruction, and cholecystectomy. Macroscopic examination of the PPPD specimen revealed a 17.0 × 2.0 mm ulcerated and infiltrative tumor located in the major ampulla (Figure 2A). In addition, a firm white lesion, 12.0 mm in diameter was detected in the duodenum (Figure 2B). Microscopic examination of the major ampulla revealed a poorly differentiated adenocarcinoma (pT2 pN1 R0) infiltrating the duodenal wall with focal necrosis (Figures 3A,B). Another lesion was identified in the minor ampulla corresponding to a well-differentiated NET (pT1 pN0 R0) 5.0 mm in diameter and without lymphovascular invasion (Figures 3C,D). The third synchronic tumor identified in the patient, a fusiform low-grade GIST, with a proliferative index of 2% (pT1 pN0 R0) was identified in the duodenal wall (Figures 3E,F). The patient was not eligible for chemotherapy or radiotherapy.

The patient reported a clinical diagnosis of NF1 since the age of 14 years and therefore, was referrd to the institutional genetic cancer risk assessment program. Physical examination identified multiple cutaneous neurofibromas and café-au-lait spots in the gluteal area, lower and upper limbs. Review of the past medical history was significant for diagnosis of viral hepatitis but she denied any previous history of pancreatitis. In addition, review of medical records and pathology reports confirmed previous occurrence of pheochromocytoma (at age 54 years), and breast cancer (first diagnosis at age 58 years and recurrence at age 61 years), all of which are features of the NF1 cancer predisposition syndrome. Review of the patient’s family history revealed that her mother also had symptoms consistent with NF1 (café-au-lait spots and neurofibromas) although no formal clinical evaluation or molecular testing had been performed (Supplementary Figure S1A).

## Materials and Methods

### Multigene Panel Testing

After signing a written informed consent for genetic analysis, a saliva sample was obtained with standard procedures using the Oragene DNA OG-500 self-collection kit (DNA Genotek, Ottawa, CA, United States) and the sample was submitted to germline genetic testing using a multigene panel test (MGPT) in a commercial laboratory (Invitae, San Francisco, CA, United States). The decision to investigate with a multigene panel was prompted by the presence of clinical features not consistent with NF1, such as pancreas divisum and by the diagnosis of five distinct primary tumors. Using Illumina technology, the presence of small variants, duplications and deletions in 29 genes (APC, ATM, BMPR1A, BRCA1, BRCA2, CDKN2A, EPCAM, MEN1, MLH1, MSH2, MSH6, NF1, PALB2, PMS2, SMAD4, STK11, TP53, TSC1, TSC2, VHL, CDK4, FANCC, PALLD, CASR, CFTR, CPA1, CTRC, PRSS1, and SPINK1) associated with hereditary pancreatic cancer and pancreatitis were assessed in the patient. All targeted regions were sequenced at a minimum of 50× depth and reads were aligned to a reference sequence (GRCh37) and with an average coverage depth of at least 350×.

### Sanger Sequencing

Sanger sequencing was performed to confirm the occurrence of the germline pathogenic variants found in MGPT testing. First, genomic DNA was extracted from patients’ peripheral blood leucocytes using FlexiGene DNA kit (Qiagen, Hilden, Germany), according to the manufacturer’s instructions. Specific primers for *NF1* exon 8 and *CFTR* intron 9/exon 10 were designed using Primer Blast^1^ and used to amplify these regions by polymerase chain reaction (Supplementary Box S1). The reference sequences used were LRG_214 and LRG_663 for *NF1* and *CFTR*, respectively. Sanger sequencing was performed on an ABI 3500 genetic analyzer (Thermo Fisher Scientific, CA, United States). Sequences were aligned to the reference sequences using CodonCode Aligner software implemented in MEGA 6.06.

## Results

Germline multigene panel testing revealed a novel heterozygous germline deletion in exon 8 of the *NF1* gene, c.838del, p.(Ile280^∗^) (*NF1* chr17:29509630, NM_000267.3) which was reported as pathogenic since it predicts a premature stop codon, resulting in the absence of translation of all NF1 protein functional domains. This variant was not previously reported in the scientific literature, public variant databases (ClinVar and LOVD) or population databases (ExAC, Abraom, and 1000 genomes). Considering that more than 4000 different *NF1* variants distributed along its 58 exons are reported to date in the ClinVar database, only a small portion are located in exon 8 (77 variants). Of these, only 16 are pathogenic. The significant allelic heterogeneity observed in NF1 renders genotype-phenotype correlations difficult, and no correlation between *NF1* variants in exon 8 and a specific phenotype have been established so far.

Additionally, one pathogenic germline variant was identified in heterozygosity in intron 9 of the *CFTR* gene, c.1210-34TG[12]T[5], (NM_000492.3). This sequence variant, also referred to as TG12-5T or T5TG12 in the literature, consists of 12TG and 5T sequence repeats on the same chromosome and, although it does not directly change the encoded amino acid sequence of the CFTR protein, it increases alternative splicing of exon 10 (referred to as exon 9 in some publications) from mRNA.

Sanger sequencing of peripheral blood DNA confirmed the germline deletion in *NF1* exon 8 c.838del, p.(Ile280^∗^) and the *CFTR* intron 9 variant, c.1210-34TG[12]T[5] (Supplementary Figure S1B,C).

## Discussion

It is well known that NF1 patients are predisposed to solid tumors, including those of the gastrointestinal tract (Agaimy et al., 2012), such as tumors arising in the ampulla of Vater and the duodenum. GIST, carcinoid tumors and other NETs are frequently reported in NF1 patients and they are considered part of the NF1 tumor spectrum, occurring in isolated manifestation or, in rare events, in synchronism with other tumors (Sørensen et al., 1986; Kim et al., 2014; Park et al., 2019). The co-occurrence of periampullary NET tumors and GIST has been proposed as highly suggestive or even pathognomonic of NF1 (Agaimy et al., 2012; Park et al., 2019; Poredska et al., 2019). LOH is a common event for the development of malignancies in NF1 patients, and has been considered by some as a prerequisite to cancer occurrence (Ruggieri and Packer, 2001). However, the development of specific tumors may require not only LOH, but also additional genetic alterations (Patil and Chamberlain, 2012).

In this report, the patient was diagnosed with multiple tumors (GIST, NET of the minor ampulla, and adenocarcinoma of the major ampulla), of which GIST and NET are likely related to NF1, a diagnosis confirmed by germline genetic testing. Among the tumors identified, GIST represents the most common gastrointestinal tumor in NF1 patients (Poredska et al., 2019), accounting for 5 to 25% of all NF1 neoplasms (Zöller et al., 1997; Miettinen et al., 2006). The last update of duodenal and periampullary tumors in NF1 patients indicates that most (60%) neoplasms arise in the duodenum, while 31% originate in the ampulla of Vater. Stratification by histology shows that GISTs correspond to 34% of duodenal tumors and the majority of ampullary tumors are neuroendocrine (40%), while only 8% are adenocarcinomas (Relles et al., 2010). Although adenocarcinomas are not usually considered part of the NF1 tumor spectrum, their occurrence should be carefully evaluated in NF1 patients. In fact, adenocarcinomas associated with NF1 seem to be rare events and have been reported in only a few studies (Deschamps et al., 2010; Tewari et al., 2014). In addition to the diagnosis of NF1, an anatomical anomaly was identified in the patient: pancreas divisum. Pancreas divisum has been reported in some NF1 patients with periampullary tumors and in most of them it is an incidental finding, with no apparent relationship with the development of neoplasia or with the syndrome itself (Waisberg et al., 2006; Bhandari et al., 2015). In general, the majority of patients with pancreas divisum do not develop symptomatic disease and there is no evidence of a direct relationship between this developmental anomaly and cancer (Ferri et al., 2019).

In addition to the germline *NF1* variant, a heterozygous germline pathogenic *CFTR* variant c.1210-34TG[12]T[5] in gene was identified. CFTR encodes the cystic fibrosis transmembrane conductance regulator, which is a membrane protein and chloride channel. Pathogenic variants in *CFTR* decrease ion channel function and cause extracellular mucus build-up; excessively thick and sticky mucus obstructs airways and pancreatic ducts, resulting in cystic fibrosis (Wang et al., 2014). Although heterozygous carriers of pathogenic *CFTR* variants do not develop cystic fibrosis, they may have an approximately 4–10-fold increased risk for pancreatitis and associated pancreatic injury due to elevated mucus levels, fibrosis, and cyst formation (Noone et al., 2001; Schneider et al., 2011; Steiner et al., 2011; Hegyi et al., 2016). On the other hand, acute and chronic pancreatitis and associated pancreatic injury are risk factors for the development of pancreatic cancer (Becker et al., 2014; Kirkegård et al., 2018). In this context, it has been reported that pancreas divisum acts as modulator of pancreatitis risk in carriers of additional genetic variants such as the one described here in the *CFTR* gene (Bertin et al., 2012; Gutta et al., 2019).

Thirteen to thirty seven percent of pancreatitis patients are heterozygous for *CFTR* mutations (Zoller et al., 2007). In a large population-based study that screened approximately 320.000 individuals for cystic fibrosis carrier status, the allelic frequency of the variant c.1210-34TG[12]T[5] in *CFTR* was 0.04 (Strom et al., 2004). The frequency of this variant in other databases is questionable, since metrics indicate poor data quality at this genomic position. Despite its high frequency in the general population, this intronic variant is a pathogenic variant, confirmed by functional studies, which show that it results in a non-functional CFTR protein through abnormal splicing (Delaney et al., 1993; Strong et al., 1993; Niksic et al., 1999; Groman et al., 2004; Bombieri et al., 2011). The polymorphic polypyrimidine (Tn) is associated with a variable efficiency of exon 10 splicing, and among the three different major variants (5T, 7T, and 9T), T5 allele is associated with a high frequency of alternatively spliced *CFTR* mRNA with loss of exon 10 (Chu et al., 1993). In a mini-gene assay, the percentage of *CFTR* mRNA without exon 10 was 54% for TG[11]T[5], 72% for TG[12]T[5] and 100% for TG[13]T[5] (Niksic et al., 1999). For these reasons, this TG[12]T[5] variant has been classified as pathogenic. The specific combination of pancreas divisum and pancreatitis was previously reported in a carrier of the same pathogenic variant (Dray et al., 2007; Montagnani et al., 2013). Moreover, several polymorphic changes in *CFTR* gene (even some with higher allele frequencies than TG[12]T[5]) have been associated with chronic pancreatitis (de Cid et al., 2010) and higher susceptibility of respiratory infections (Polgreen et al., 2018).

In this case report, we describe an unusual clinical presentation in an NF1 patient and emphasize the importance of a comprehensive analysis of potential risk factors in situations with atypical and/or complex phenotypes. In this particular case, the combination of factors associated with pancreatitis (pancreas divisum and the genetic change in *CFTR*) and the pathogenic variant in *NF1* may have had a synergistic role to increase the risk for occurrence of multiple tumors. With the ever increasing availability of MGPT, identification of individuals with more than one pathogenic germline variant is expected to increase and the critical review of these variants in terms of their causal effect will be key to determine patient care and follow-up recommendations.

## Conclusion

The current report, to our knowledge, is the first clinical description of an NF1 patient with pancreas divisum and multiple periampullary tumors. The occurrence of a pathogenic variant in *NF1* and another pathogenic variant in *CFTR* in the same patient is also reported for the first time. The identification of two germline variants and a developmental anomaly in this patient may explain the unusual and more severe findings. However, whether the combination these factors increases risk for multiple tumors in an additive manner remains to be determined. The particular genetic status of this patient will require careful surveillance for lifetime cancer risk as well as appropriate genetic counseling for her relatives and underscores the importance of comprehensive genetic testing in patients with complex phenotypes.

## Ethics Statement

Written informed consent was obtained from the patient for publication of this case report and accompanying images. The study was conducted in accordance with the Declaration of Helsinki and has been approved by the Scientific and Research Committee of Hospital de Clínicas de Porto Alegre (protocol number 13-0260).

## Author Contributions

CG conceived the work and conception design of the case report. CG and CR design of the draft the manuscript. CG, CR, and CN were involved in patient recruitment. SM, LA, CN, VB, AO, and PA-P provided clinical data, were directly involved in the clinical follow-up and helped to draft the manuscript. LA and SM carried out and interpreted the imaging studies. All authors revised the manuscript critically, contributed with interpretation of the findings and gave final approval of the version to be published. PP supervised the work.

## Conflict of Interest

The authors declare that the research was conducted in the absence of any commercial or financial relationships that could be construed as a potential conflict of interest.

## Footnotes

1 http://www.ncbi.nlm.nih.gov/tools/primer-blast/

## Supplementary Material

FIGURE S1 Pedigree of a family carrying variant *NF1* c.838del and Sanger sequencing electropherogram from heterozygous germline variants. **(A)** Pedigree of the *NF1* c.838del heterozygous germline variant. The arrow indicates the proband; current age is indicated in parenthesis. Sanger sequencing electropherogram from proband demonstrating **(B)** *NF1* Exon 8 heterozygous germline variant (c.838del) and **(C)** *CFTR* Intron 9 heterozygous germline variant c.1210-34TG[12]T[5].

BOX S1 Primers and PCR conditions.
