# Supplementary figures and images for: Synchronous Periampullary Tumors in a Patient With Pancreas Divisum and Neurofibromatosis Type 1
Source: Front Genet. 2020 Apr 28;11:395. doi: 10.3389/fgene.2020.00395 (PMC7212385; doi:10.3389/fgene.2020.00395)

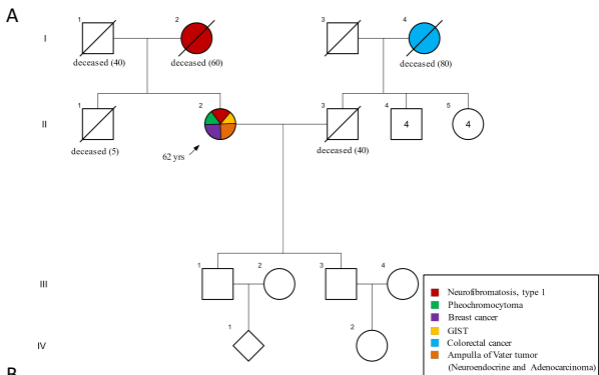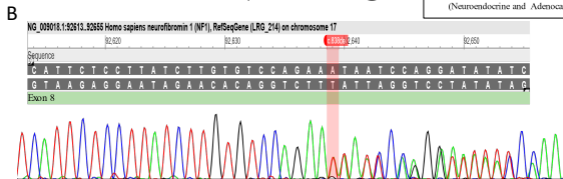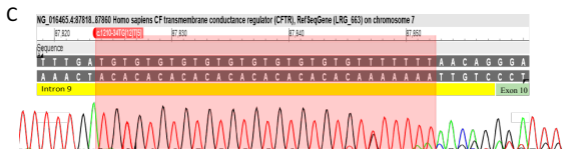

Supplement: FIGURE S1 — Pedigree of a family carrying variant NF1 c.838del and Sanger sequencing electropherogram from heterozygous germline variants. (A) Pedigree of the NF1 c.838del heterozygous germline variant. The arrow indicates the proband; current age is indicated in parenthesis. Sanger sequencing electropherogram from proband demonstrating (B) NF1 Exon 8 heterozygous germline variant (c.838del) and (C) CFTR Intron 9 heterozygous germline variant c.1210-34TG[12]T[5]. [file Image_1.pdf]
