# Supplementary material for: Synchronous Periampullary Tumors in a Patient With Pancreas Divisum and Neurofibromatosis Type 1
Source: Front Genet. 2020 Apr 28;11:395. doi: 10.3389/fgene.2020.00395 (PMC7212385; doi:10.3389/fgene.2020.00395)
Supplement: BOX S1 — Primers and PCR conditions. [file Table_1.DOCX]

**Supplementary Box 1.** Primers and PCR conditions.

| ***CFTR* intron 9- exon 10** | ***NF1* exon 8** |
| --- | --- |
| **Primers** |  |
| Foward - 5’atgtcctctagaaaccgtatgc 3’ | Foward - 5’ tgccagggattttgttcctatct 3’ |
| Reverse - 5’ gtagtgctggaaggtatttttggaga 3’ | Reverse - 5’ agcctaaagtaatacacaccttgag 3’ |
|  |  |
| **PCR conditions (per sample)** |  |
| Taq Platinum Buffer 10x – 2,0ul | Taq Platinum Buffer 10x– 2,0ul |
| dNTP 50mM – 0,8ul | dNTP 50mM - 0,8ul |
| MgCl_2_ 25mM – 1,0ul | MgCl_2_ 25mM – 1,0ul |
| Primer 10mM (F e R) - 0,5ul each | Primer 10mM (F e R) - 0,5ul each |
| Taq Platinum - 0,5U | Taq Platinum - 0,5U |
| DNA - 10ng | DNA - 10ng |
| Q.s.p. - 20ul | Q.s.p. - 20ul |
|  |  |
| **Thermocycling conditions** |  |
| 95ºC – 5min  94ºC – 30seg  65ºC – 60seg 30 cycles  72ºC – 60seg  72ºC – 10min  4ºC – ∞ | 95ºC – 5min  94ºC – 30seg  62ºC – 60seg 30 cycles  72ºC – 60seg  72ºC – 10min  4ºC – ∞ |
